# The doctor is naked – and the blindness of our medical education

**DOI:** 10.47626/2237-6089-2023-0768

**Published:** 2025-09-18

**Authors:** Fábio Augusto do Prado Marmirolli, Thiago Marques Fidalgo, Fernanda Gonçalves Moreira

**Affiliations:** 1 Departamento de Psiquiatria Escola Paulista de Medicina Universidade Federal de São Paulo São Paulo SP Brazil Departamento de Psiquiatria, Escola Paulista de Medicina, Universidade Federal de São Paulo (UNIFESP), São Paulo, SP, Brazil.

The discussion about teaching human sciences in medical education and elaborating on a humanistic medical education in Brazil has recently resurfaced among doctors, professors, and the general population due to recent events, like the so-debated episode of sexual harassment featured by medical students during a sports competition in 2023.^1^ A group of male students ran naked into a volleyball court during a feminine game part of the college games. Some male students simulated masturbation in public, in an unmistakable gesture of harassment against the female players.

The sexist culture in medical education is not exclusive of the Brazilian reality. A study with 524 students from four medical schools in the United States showed 38.5% of students reported sexual harassment by a fellow student. Sexist gender and crude gender harassment were the most common ones.^2^ Another research with 1,059 medical students in Switzerland showed associations between being targeted by harassment/sexism and risk of depression, suicidal ideation, and anxiety.^3^ Both studies found a higher prevalence of sexism and harassment in medical school’s final/clinical years, which may suggest there is something in the medical education process that influences this behavior. It is interesting to compare that both United States and Switzerland have much lower scores on Gender Inequality Index (GII)^4^ – a scale that reflects gender disadvantage around the world – than Brazil (0,179 and 0,018 compared with 0,390 in 2021, respectively), which may represent an even worse scenario for female Brazilian medical students. National data shows that only in the first semester of 2023 more than 34 thousand rapes of girls and women were reported in Brazil.^5^

The presence of gender violence may also reflect the so-called hidden curriculum in medical schools and the students’ poor mental health. A recent British article showed medical students have more chances of experiencing a large number of mental health problems than students of other subjects.^6^ This same article describes that a hidden curriculum in medical schools may cause a decline in empathy as students advance through the course and highlights other risk factors for a poorer mental health status, such as a competitive learning environment and stressful workload, leading to cynicism and emotional distance.^6^ Also, medical students in the United Kingdom had a 62% prevalence of hazardous consumption of alcohol, a problem also present in Brazilian medical students.^7^ Although, another British study showed that young adults with a higher education level had lower average psychosocial distress compared to young adults out of university.^8^

An editorial from Revista Brasileira de Educação Médica – the official journal of the Brazilian Association of Medical Education (Associação Brasileira de Educação Médica [ABEM]) – from 1980 already called attention to the lack of humanization in medical curricula and pointed out the subject of medical psychology as a pathway for change and improvement.^9^ Forty-three years later, we again point in the same direction, as the message continues to be extremely necessary, as this harassment episode clearly shows.

The medical psychology discipline is a privileged space in the medical education curriculum to discuss and reflect on the subjectivity of individuals and human relationships.^10^ The object of the discipline goes beyond the teaching of the doctor-patient relationship but also encompasses the adaptation of the student to “the school, the career and life,”^11^ the better understanding of themselves and others, which may auxiliate in healthier relations in campus, through a broader exercise and ability of empathy. Medical psychology is also aligned with the 2014 National Curriculum Guidelines, which state that medical courses need to train “humanistic, critical, and reflexive” medical doctors.^12^ However, the reality for many medical courses in Brazil is far different.

The authors of this editorial are now conducting a study to assess if the 388 medical schools in Brazil include medical psychology concepts in their pedagogical projects and how they propose to do so. The authors researched the official websites of every Brazilian medical school. If the pedagogical project was unavailable online, the institutions were contacted via e-mail at least three times with a solicitation for the desired document. After this procedure, 188 pedagogical projects were obtained from all Brazilian states. Initially, each document was searched to find if and where the term *psicologia médica* (medical psychology) was present in the text. As this study used only publically available documents, we did not need ethical approval. Preliminary results show that, from those, 70 do not even cite this term. Although it is unclear yet if the contents from medical psychology are present in these medical schools using another terminology – the research will clarify this point by expanding the terms search – it calls our attention, especially in the face of the recent events.

Are we going back to a strict biological teaching of medicine, ignoring subjectivity and human relationships in the structure of the medical curriculum? A review from the Lancet about best practices towards gender equality listed changes in curriculum as an organizational intervention needed for better equality.^13^ However, there is much more research on diagnosing inequalities in medical scenarios than intervention studies on the problem. A review aiming to evaluate programs to prevent mistreatment of medical trainees in the United States found just ten articles about intervention strategies with a low overall quality.^14^

Escola Paulista de Medicina at Universidade Federal de São Paulo pioneered medical psychology education in Brazil in 1956. The institution integrates medical psychology with various medical disciplines, fostering a holistic approach. In the 1st year, “medical psychology, health, and society” explores illness concepts and societal impacts, uniting medical psychology, collective health, and human sciences. The 2nd year’s “medical psychology” teaches personality development, life cycles, empathy, and understanding through narrative medicine. The 3rd year’s “semiology of human relationships” uses art to develop sensibility and humanistic qualities in future doctors. In the 4th year, “integral attention to women and children health” emphasizes communication skills and grief management during the psychiatry internship. Innovative teaching methods are employed in most of these programs. In the 5th year, students dedicate a month to mindfulness techniques for improved patient care through self-care and self-observation.

Although all the efforts carried out, the improvements in teaching abilities of empathy and communication, as well as the sensibilization of the students in the care of others, are not enough to change the culture of sexism, stigma, and competitiveness in medical schools, as well to improve medical students mental health, without the implementation of institutional and government policies addressing mental health care of this population and strategies to reduce discrimination and violence in the university’s environment. Mindfulness-based interventions, reflection groups, and curriculum changes are strategies used worldwide to improve students’ well-being. Still, most are optional to students isolated in just one institution.^15^ Despite the significant number of medical schools in Brazil, only a few have mental health services specifically designed for students.^16^

Medical schools are still advancing and looking for new models of medical education, but always keeping in mind the importance and relevance to patients and society of an even more human teaching of Medicine. As the recent events demonstrate, we are not always being successful. However, all efforts must be made to develop an effective manner of doing so. Perhaps, one day, empathy and concern for others will be more present in medical schools than anthems about rape and misogyny.^1^
